# Water-Soaking Pretreatment for Enhanced Performance and Heavy Metal Immobilization in Alkali-Activated Pyrolysis MSWIFA Materials

**DOI:** 10.3390/ma18194520

**Published:** 2025-09-28

**Authors:** Shengyu Zhong, Liang Shen, Wanlan Xu, Yi Fang, Yunfeng Pan

**Affiliations:** 1 School of Civil Engineering and Architecture, Zhejiang Sci-Tech University, Hangzhou 310018, China; 2023210802017@mails.zstu.edu.cn (S.Z.);; 2 Hangzhou Huihong Environmental Technology Co., Ltd., Hangzhou 310058, China; 3 College of Civil and Transportation Engineering, Hohai University, Nanjing 210098, China

**Keywords:** MSWIFA, pyrolysis fly ash, alkali-activated materials, compressive strength, heavy metals solidification

## Abstract

This study demonstrates that synergistic pyrolysis and water-soaking pretreatment transforms municipal solid waste incineration fly ash (MSWI FA) into high-performance alkali-activated materials when combined with ground granulated blast furnace slag (GGBS). Pyrolysis reduced chlorine content by 94.3% and increased reactive components by 44.4%, thereby shifting hydration products from Friedel’s salt to ettringite (AFt). Subsequent water-soaking eliminated expansion-causing elemental aluminum, liberating activators for enhanced reaction completeness (29% higher cumulative heat release) and enabling a denser matrix with 71.5% harmless pores (<20 nm). The dual-treated FA (T-PFA) achieved exceptional mechanical performance—295.6% higher 56-day compressive strength versus untreated FA at a 1:1 ratio—while reducing porosity by 29.1% relative to pyrolyzed-only FA. Despite 22–38% increased total heavy metal content post-pyrolysis, matrix densification and enhanced C-A-S-H/AFt formation reduced Cr/Cd/Cu/Pb leaching by 11.3–66.7% through strengthened physical encapsulation and chemisorption, with all leachates meeting stringent HJ 1134-2020 thresholds. This integrated approach provides an efficient, environmentally compliant pathway for MSWI FA valorization in low-carbon construction materials.

## 1. Introduction

Municipal solid waste incineration fly ash (MSWIFA), a hazardous byproduct derived from urban waste incineration, presents a growing environmental challenge due to accelerating urbanization. Annual MSWIFA production is substantial, exceeding 20 million tons in China alone during 2023 (China Statistical Yearbook). Classified as hazardous in many countries, MSWIFA contains heavy metals (HMs) and toxic organic pollutants, meaning it poses significant environmental risks if improperly managed [1]. Stabilization/solidification (S/S) using cementitious materials offers an efficient and environmentally friendly treatment method for these toxic substances [2]. Cementitious matrices immobilize HMs and organic toxins through physical encapsulation, ion exchange by hydration products, and chemical adsorption, thereby mitigating environmental hazards [3]. However, conventional cement, the most widely used binder, involves energy-intensive production with high greenhouse gas emissions [4]. Furthermore, MSWIFA’s high chlorine (Cl) content—resulting from significant kitchen waste proportions in municipal solid waste—accelerates steel reinforcement corrosion in concrete [5,6]. Additionally, MSWIFA’s inherently low content of reactive materials limits its incorporation rate in cement-based systems [7]. Consequently, researchers are exploring pretreatment technologies for fly ash combined with alkali activation to develop alternative alkali-activated cementitious materials [8,9,10,11].

Alkali-activated cementitious material (AAM) is an inorganic binder formed by the reaction of an alkaline solution (e.g., sodium hydroxide or sodium silicate) with aluminosilicate-rich precursors [12]. AAMs offer dual advantages: they effectively utilize waste-derived components like CaO, SiO_2_, and Al_2_O_3_, and, unlike Portland cement, their production avoids calcination, significantly reducing associated greenhouse gas emissions and energy consumption [13]. Consequently, AAM is widely regarded as a promising cement alternative [14]. Similar to cement, AAM can effectively stabilize/solidify heavy metals through multiple mechanisms [15,16,17]. Fly ash, containing essential components like CaO, SiO_2_, and Al_2_O_3_, serves as a key precursor for AAM preparation [18]. However, the performance of fly ash-based AAM (FA-AAM) is highly dependent on the ash’s chemical composition and reactivity [19]. Specifically, the proportion of active components and their reactivity directly govern the hydration kinetics and microstructure development under alkali activation, ultimately determining the FA-AAM’s overall performance [15,20]. Optimizing fly ash selection and pretreatment is therefore critical for enhancing FA-AAM.

Traditional pretreatment methods—including water washing [9], alkali washing [21], acid washing [22], and chemical chelation [23,24]—aim to enhance fly ash reactivity and remove chlorine, heavy metals (HMs), and soluble salts. However, these methods introduce significant drawbacks, such as the generation of secondary wastewater requiring treatment and potential environmental safety concerns, leading to additional resource burdens and pollution risks. Furthermore, they are generally ineffective at removing persistent organic toxins (e.g., dioxins and furans) [25], whose health and environmental hazards necessitate attention during fly ash resource utilization.

In contrast, fly ash pyrolysis detoxification technology involves heating MSWIFA in a sealed, low-oxygen environment (300–700 °C) [26]. This process decomposes organic compounds and volatile inorganics into collectible gases/liquids, which can be utilized as fuel, achieving detoxification. Carbon addition during pyrolysis accelerates HM speciation transformation and organic compound decomposition. Under high-temperature, reducing conditions, aluminum oxide and chlorides react reductively (e.g., with carbon), generating elemental aluminum (Al) and chlorine gas (Cl_2_), effectively removing chlorine. However, this process introduces reactive Al. When incorporated into AAM, this elemental aluminum reacts with the alkaline activator to produce hydrogen gas (H_2_), the primary cause of detrimental expansion in FA-AAM [20]. This expansion compromises mechanical properties, HM stabilization, and long-term durability.

While pyrolysis effectively removes organic pollutants and modifies physicochemical properties, enhancing fly ash safety and suitability as an AAM precursor, the associated Al-induced expansion poses a significant challenge. Research on AAM performance using pyrolyzed fly ash or pyrolyzed ash with subsequent Al^0^ removal remains limited.

Therefore, this study prepares AAMs using GGBS blended with three fly ash variants: untreated, pyrolyzed, and pyrolyzed–water-soaked (designed to remove elemental Al generated during pyrolysis). We comprehensively compare the mechanical properties, hydration kinetics, microstructure, and HM stabilization performance of these AAMs. This work systematically investigates the influence of (1) pyrolysis pretreatment, (2) post-pyrolysis elemental Al removal, and (3) precursor composition on AAM performance. The findings provide essential guidance for utilizing pyrolyzed fly ash in alkali-activated systems and elucidate the potential advantages of both pyrolyzed and pyrolyzed–water-soaked fly ash for developing sustainable AAMs, thereby advancing fly ash resource utilization.

## 2. Materials and Methods

### 2.1. Raw Materials

Ground granulated blast furnace slag (GGBS), classified as S95-grade, was sourced from Jingye Steel Plant (Shijiazhuang, China). This white powder exhibited a specific surface area of 400 m^2^/kg and a density of 2.83 g/cm^3^. Municipal solid waste incineration fly ash (MSWIFA) was obtained from a waste incineration facility in Fuyang, Hangzhou (Huihong Environmental Technology, Hangzhou, China). Two variants were investigated: raw fly ash (RFA) and pyrolyzed fly ash (PFA). PFA is produced by low-temperature pyrolysis at 550 °C for 6 h through RFA, effectively removing organic toxins including dioxins.

The alkali activator consisted of NaOH (used as a prepared solution in the experiments, purity > 98%, Shanghai McLin Biochemical Technology Co., Ltd., Shanghai, China) and sodium silicate solution (analytical reagent; SiO_2_: 26.5%, Na_2_O: 8.3%; Shandong Yousuo Chemical Technology Co., Ltd., Jinan, China). These components were blended to achieve a modulus (SiO_2_/Na_2_O molar ratio) of 0.9 and an alkali equivalent (Na_2_O content) of 5% by mass of the binder. The selected modulus of 0.9 and Na_2_O content of 5% were based on preliminary tests and literature precedent [27,28], aiming to balance workability, reaction kinetics, and mechanical performance. Excessively high values led to impractical setting behavior, while lower values resulted in poor early strength and efflorescence.

Table 1 presents the chemical compositions of raw materials determined by X-ray fluorescence (XRF). The combined content of reactive components (CaO + SiO_2_ + Al_2_O_3_) was 45.02% for RFA and 65.01% for PFA, confirming their suitability as supplementary cementitious materials. Pyrolysis treatment increased the reactive components by 44.4% while reducing the chlorine content by 94.3%, demonstrating significant pyrolysis-induced physicochemical transformations.

Table 2 presents the TOC (Total Organic Carbon) test results for RFA and PFA. The significantly higher TOC value in the RFA indicates a substantial content of unburned carbon, which is known to adversely affect hydration by adsorbing water and chemical admixtures. The marked reduction in TOC for the PFA (from 1.629% to 0.620%) confirms that the pyrolysis treatment effectively removed a large portion of the unburned carbon and volatile organic matter. This reduction in organic content, corroborated by the XRF results, supports our conclusion that the pyrolysis process primarily facilitated the removal of organic matter and promoted the decomposition of certain compounds (e.g., chlorides, hydroxides), thereby enhancing the reactivity of the PFA.

**Figure 1:**
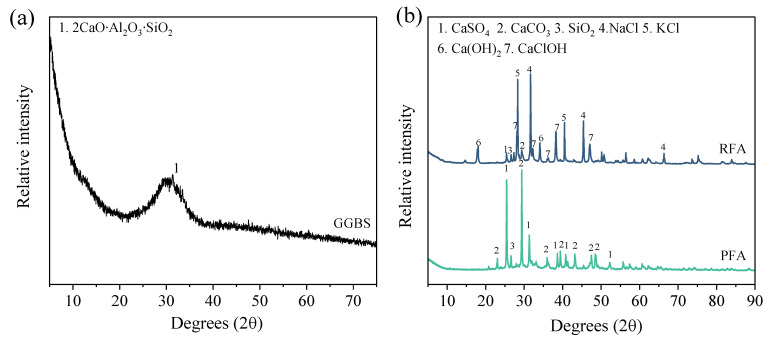
XRD patterns of raw materials: (**a**) GGBS; (**b**) RFA and PFA.

Figure 1 presents the XRD patterns of the raw materials. GGBS exhibits broad amorphous ‘halo peaks’ centered around 30°, characteristic of its predominantly glassy structure. Distinct crystalline phase differences are evident between the MSWIFA variants. RFA displays prominent diffraction peaks corresponding to chloride salts, attributable to the high proportion of food waste in the source material. In contrast, PFA shows a significant reduction in chloride-related peaks and the emergence of distinct sulfate diffraction peaks. This transformation results from pyrolysis-induced dechlorination, where chlorides are volatilized, while sulfate ions combine with available metal cations to form stable sulfate phases. The presence of these chloride and sulfate phases is expected to significantly influence subsequent hydration reactions and the formation of reaction products [5,18].

### 2.2. Treatment of Pyrolyzed Fly Ash

The incorporation of pyrolyzed fly ash (PFA) in AAM induces significant expansion, adversely affecting mechanical properties and increasing the risk of HM leaching. Substantial evidence attributes this expansion to the reaction between elemental aluminum (Al^0^) present in PFA and hydroxide ions (OH^−^) from the alkaline activator, as described by Equation (1). This reaction generates hydrogen gas (H_2_), causing volumetric expansion within the matrix [29,30]:

(1) $$2Al+2{OH}^{-}+6H_{2}O\to 2Al{\left(OH\right)}_{4}^{-}+3H_{2}↑$$

Consequently, pretreatment to eliminate reactive Al^0^ is essential when utilizing PFA in AAM formulations. Given fly ash’s inherent strong alkalinity (aqueous solution pH > 10) [31], a water-soaking pretreatment leveraging this alkalinity was employed. The removal of metallic aluminum (Al^0^) is achieved through its corrosion and subsequent dissolution in the alkaline aqueous environment during the soaking process. As indicated by Equation (1), Al^0^ reacts with water and hydroxyl ions (OH^−^) to produce hydrogen gas and soluble aluminate species (Al(OH)_4_^−^) [29,32]. This reaction is thermodynamically favorable in a high-pH environment. The resulting material is designated Treated-Pyrolyzed Fly Ash (T-PFA).

The specific treatment procedure is as follows: Firstly, PFA is soaked in deionized water at a solid-to-liquid ratio of [1:5] (kg/L) for 72 h, without changing the water during the soaking process. Then, the slurry (including both solids and soaking water) was dried at 105 °C for 24 h. After drying, treated ash was mixed with deionized water at a 1:5 (kg/L) ratio. The reaction was deemed complete when the supernatant pH stabilized above 7 and remained constant with prolonged soaking time, indicating consumption of reactive Al^0^.

### 2.3. Mix Design

The experimental matrix comprised two MSWIFA: GGBS mass ratios (1:1 and 3:1). Paste specimens (40 mm × 40 mm × 4 mm) were prepared [33]. A water-to-cementitious materials ratio (w/cm) of 0.4 was maintained [34], with an alkaline activator modulus (SiO_2_/Na_2_O) of 0.9 and an alkali equivalent (Na_2_O mass%) of 5%.

Mixing was carried out in a Hobart mixer (Hobart Corporation, Troy, OH, USA) as follows: the precursor materials were dry-mixed for 30 s, followed by mixing for another 2 min after adding the well-prepared alkaline activator (before 24 h). After a 30 s rest, mixing resumed for another 2 min. The fresh paste was cast into molds, vibrated for 10 s, sealed with plastic film, and left at ambient conditions for 24 h. Specimens were demolded, thermally cured at 80 °C for 24 h (to mitigate the autogenous shrinkage and accelerate the strength development [27]), cooled to room temperature, then transferred to a standard curing room. Mechanical tests were performed on specimens aged 3, 7, 28, and 56 days.

Table 3 lists all mix proportions (solid content). In each Mix ID, with “S,” “R,” “P,” and “TP” indicating slag (GGBS), raw fly ash (RFA), pyrolyzed fly ash (PFA), and treated pyrolyzed fly ash (T-PFA), respectively. No number or the number 3 indicate MSWIFA/GGBS ratios of 1:1 and 1:3. In addition, AAM pastes were prepared followed Table 2 for compressive strength, XRD, Thermogravimetric Analysis (TGA), Scanning Electron Microscope (SEM), Mercury Intrusion Porosimetry (MIP), and leaching toxicity of HMs.

### 2.4. Tests Methods

#### 2.4.1. Mechanical Strength

Paste mixtures were tested at curing ages of 3, 7, 28, and 56 days. Compressive strength tests were performed using a Hua-nan HYZ-300 (10) machine (South China Instrument and Equipment Co., Ltd., Shaoxing, China), following the GB/T 17671-2021 standard [33], with a loading rate of 2.4 kN/s. For each mix, six specimens were tested in compression, and the reported strengths are the average values of the six compressive results.

#### 2.4.2. Isothermal Calorimetry

The hydration heat evolution of paste mixtures was monitored for 72 h at 23 °C using a Calmetrix I-Cal 4000 calorimeter (Gangyuan Testing Instrument Factory, Tianjin, China) (ASTM C1679-17 [35]). Heat flow and cumulative heat were normalized based on the total mass of the precursor.

#### 2.4.3. X-Ray Diffraction (XRD)

The 28-day-cured paste specimens were immersed in 99% isopropanol for at least 72 h to halt hydration, followed by oven-drying at 60 °C for 24 h. The dried samples were then milled to <0.75 mm and analyzed using a Bruker D8 Advance diffractometer (Bruker Corporation, Billerica, MA, USA) in the 2θ range of 5–75° to identify crystalline phases.

#### 2.4.4. TGA

To quantify hydration products, the 28-day-cured paste was solvent-exchanged in 99% isopropanol for at least 72 h, oven-dried at 60 °C for 24 h, and ground to a particle size of <0.75 mm. About 15 mg of the sample was heated from 30 °C to 1000 °C at a rate of 10 °C/min under N_2_ using a Netzsch TGA 209 F1 (Netzsch Group, Bavaria, Germany).

#### 2.4.5. SEM

The dried and hardened paste samples were crushed, and thin flat slices were selected. After 30 s of vacuum gold coating, they were examined using a scanning electron microscope (Germany ZEISS Sigma 360, Oberkochen, Germany) at a 15 kV electron beam voltage to study the impact of water treatment on the microstructure of the matrix.

#### 2.4.6. MIP

Fragments (4–60 mm) of 28-day-cured paste were treated with isopropanol for 7 days, then oven-dried at 60 °C until a constant mass was achieved. The pore-size distribution and total porosity were determined using an AutoPore 9500 porosimeter (Micromeritics Instrument Corporation, Norcross, GA, USA) at pressures up to 210 MPa.

#### 2.4.7. Leaching Toxicity of Heavy Metals

The total heavy metal content in RFA, PFA, and T-PFA was analyzed using the microwave digestion method (China, HJ-781-2016 [36]). TCLP (Toxicity Characteristic Leaching Procedure) leaching tests (EPA SW-846 Method 1311 [37]) were conducted on crushed 28-day paste (<9.5 mm). Samples were mixed with acetic acid (L/S = 20:1 L/kg) at 30 rpm for 18 h, then filtered (0.45 µm), acidified (5% HNO_3_), and analyzed for Zn, Pb, Cu, Cd, and Cr concentrations using ICP-OES.

## 3. Results and Discussion

### 3.1. Effectiveness of Water-Soaking Treatment for Expansion Control

**Figure 2:**
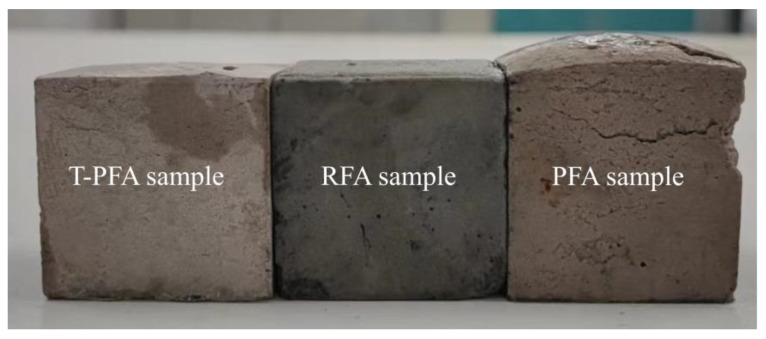
Visual comparison of AAM specimens: T-PFA sample (**left**), RFA sample (**center**), and PFA sample (**right**). Note the significant expansion and cracking in the PFA sample.

Visual evidence supporting this finding is presented in Figure 2, showing AAM specimens prepared with T-PFA, RFA, and PFA at a 1:1 fly ash/GGBS mass ratio. Specimens containing PFA exhibit severe expansion and associated cracking, consistent with Al^0^-induced hydrogen gas generation. In contrast, specimens incorporating T-PFA (water-soaked PFA) display no noticeable surface cracks and maintain a volume comparable to RFA-based specimens. This confirms the efficacy of the water-soaking pretreatment in removing expansion-causing Al^0^ from PFA.

### 3.2. Physicochemical Characterization of T-PFA

**Figure 3:**
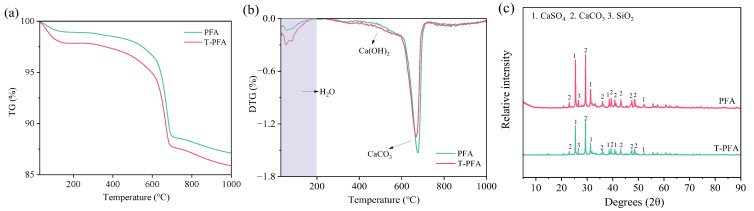
Characterization of PFA and T-PFA: (**a**) TG; (**b**) DTG; (**c**) XRD patterns.

Figure 3 presents TG (Thermogravimetry), DTG (Derivative Thermogravimetry), and XRD analyses comparing PFA and T-PFA. The TG/DTG results (Figure 3a,b) reveal a significant increase in mass loss for T-PFA below 200 °C, primarily attributable to the evaporation of free and weakly bound water absorbed during soaking. A slight increase in mass loss around 450 °C and a concurrent decrease around 750 °C (associated with carbonate decomposition) in T-PFA suggest partial dissolution and reaction of carbonates (e.g., CaCO_3_) with water, potentially forming calcium hydroxide (Ca(OH)_2_) during soaking.

XRD analysis (Figure 3c) confirms no new crystalline phases formed in T-PFA. However, a distinct reduction in the intensity of the calcium carbonate (CaCO_3_) diffraction peak aligns with the DTG observations, supporting the hypothesis of carbonate consumption via dissolution/reaction during water treatment.

XRF compositional analysis (Table 4) further elucidates the effects of soaking. While most oxide contents remain largely unchanged, a notable increase in sulfur trioxide (SO_3_) content is observed in T-PFA (19.21 wt.% vs. 17.57 wt.% in PFA). This increase likely stems from dissolved sulfides (e.g., S^2−^) oxidizing upon exposure to atmospheric oxygen during soaking, forming stable sulfate species (SO_4_^2−^) that precipitate upon drying that are readily quantified by XRF. A minor reduction in chloride (Cl) content is also noted.

The above results indicate that water-soaking treatment successfully consumes expansion-causing elemental aluminum in PFA, as evidenced by pH stabilization, visual specimen integrity, and the elimination of expansion. The primary physicochemical changes induced in PFA are (1) increased moisture content, (2) partial consumption of carbonates, and (3) oxidation of sulfides to sulfates, leading to a measurable increase in SO_3_ content. No significant alterations to other major oxide components were detected.

### 3.3. Compressive Strength Development

**Figure 4:**
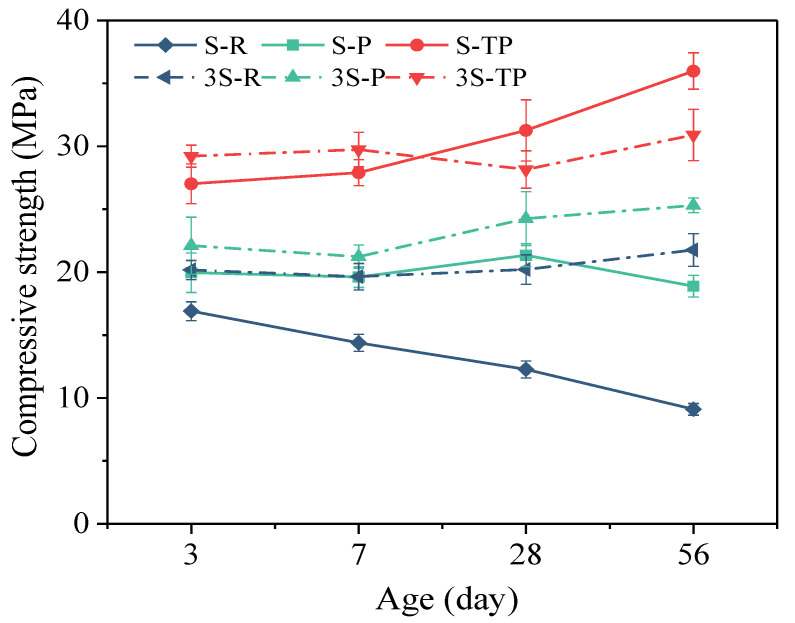
Compressive strength development of AAM paste specimens.

Figure 4 shows the compressive strength development of paste specimens, which was strongly influenced by both the MSWIFA/GGBS ratio and the type of fly ash. This trend is consistent with the compositional data (Table 1 and Table 3), where GGBS contains 87.2% reactive phases (CaO + SiO_2_ + Al_2_O_3_), a figure substantially higher than for RFA (45.0%) and PFA/T-PFA (~64–65%). Consequently, GGBS acted as the primary contributor to strength development.

Specimens incorporating RFA exhibited significantly lower compressive strength than those with PFA or T-PFA, mainly due to RFA’s low reactive component content. In mixtures with low GGBS content, RFA’s high chloride concentration further competed with reactive phases for the alkaline activator, inhibiting the setting of alkali-activated materials (AAMs) and causing matrix discontinuity [38,39]. This, coupled with the insufficient supply of reactive phases, hindered stable strength development and led to noticeable late-age strength decline.

At a 1:1 MSWIFA/GGBS ratio, PFA specimens reached 18.88 MPa at 56 days, representing a 107.5% increase over RFA specimens (9.1 MPa). However, in mixtures with higher PFA contents, residual elemental aluminum caused expansion, creating a porous microstructure that slightly reduced late-age strength (56 days). Notably, at the same 1:1 ratio, T-PFA specimens achieved higher 28-day strength than those with a 1:3 ratio, indicating superior early performance. Ultimately, T-PFA specimens at the 1:1 ratio reached 36.0 MPa at 56 days—a 295.6% increase over RFA specimens at the same ratio. This substantial improvement was attributed to the removal of expansion-inducing elemental aluminum, which significantly densified the matrix and produced a more compact structure. Moreover, eliminating competitive aluminum reactions increased activator availability for the hydration of T-PFA’s reactive phases, promoting the formation of abundant hydration products and greatly enhancing strength.

The compressive strength results demonstrate that pyrolysis pretreatment significantly enhances the reactivity of the fly ash, leading to a notable improvement in the mechanical properties of the resulting alkali-activated materials. This is consistent with previous studies indicating that thermal treatment can enhance the reactivity of MSWIFA. The removal of chloride during pyrolysis also likely mitigates its potential inhibiting effect on hydration, thereby supporting stronger matrix development.

Furthermore, the additional water soaking step applied to PFA (yielding T-PFA) led to a further marked increase in strength. This aligns with our hypothesis that removing soluble aluminum compounds via leaching prevents the premature formation of poorly crystalline aluminates, thereby allowing alkalis to more effectively activate the precursor materials and facilitate the formation of a more cohesive and denser C-A-S-H gel network. The consequent microstructural refinement is corroborated by the MIP and SEM results.

These findings confirm the synergistic effect of the combined pyrolysis–water-soaking treatment in transforming MSWI fly ash into a highly effective precursor for alkali-activated materials. The fact that the 28-day strength of T-PFA samples exceeded 20 MPa—meeting the standard for non-load-bearing masonry units—highlights the practical potential of this treatment for producing precast construction elements. Future work will focus on optimizing the treatment parameters for different ash sources and exploring long-term durability under various environmental conditions to support broader engineering applications.

### 3.4. Hydration Heat Evolution

**Figure 5:**
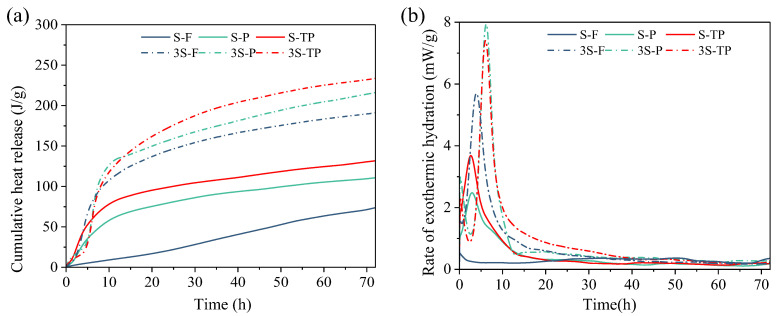
Hydration kinetics of AAMs incorporated with MSWIFA: (**a**) cumulative heat; (**b**) hydration heat rate.

Figure 5 shows the hydration heat evolution of pastes with different MSWIFA/GGBS ratios. The heat flow rate curves (Figure 5b) indicate that mixtures with a 1:3 ratio exhibited a markedly higher maximum exothermic peak than those with a 1:1 ratio, reflecting greater reaction activity and accelerated early hydration kinetics. This enhancement is attributed to the higher GGBS content and greater proportion of reactive phases in the 1:3 blends. The cumulative heat release curves (Figure 5a) corroborate this trend: within 72 h, cumulative heat release for the 1:3 ratio was consistently higher than that for the 1:1 ratio across all fly ash types.

Hydration behavior varied notably with fly ash treatment. For RFA at the 1:1 ratio, a pronounced initial dissolution peak was observed upon water contact (Figure 5b), followed by the absence of significant main hydration peaks and only minor exothermic signals, likely associated with NaCl and Friedel’s salt formation. This suppression of the primary hydration reactions is attributed to RFA’s high Cl^−^ content, which competes with aluminates for activators and impedes proper setting [38].

In contrast, PFA specimens exhibited substantially higher maximum heat flow and cumulative heat release than RFA, reflecting enhanced reactivity due to pyrolysis-induced enrichment of reactive phases. T-PFA showed the highest cumulative heat release overall. Removal of elemental aluminum eliminated competitive expansion reactions, freeing activators to participate in productive hydration with T-PFA’s reactive components. This synergistic effect explains the superior hydration reactivity of T-PFA. Given the constant proportions of fly ash and GGBS across all mixes, the observed increase in heat release is attributed to an elevated degree of reaction resulting from the thermal pretreatment, rather than a change in the contribution of GGBS. The progressive increase in cumulative heat release (RFA < PFA < T-PFA) confirms that pyrolysis followed by water soaking markedly improves the reactivity of MSWIFA in alkali-activated systems.

### 3.5. Phase Evolution and Thermal Analysis of Hydration Products

**Figure 6:**
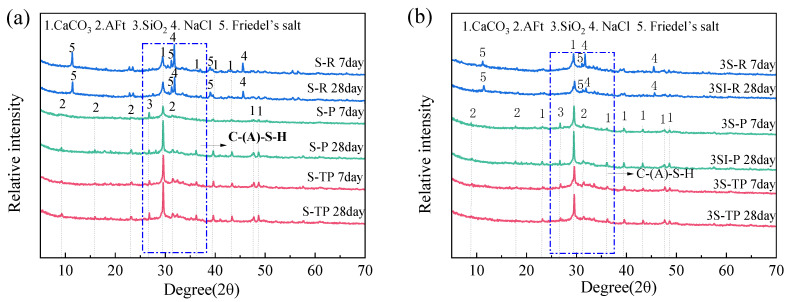
XRD patterns of hardened paste specimens: (**a**) MSWIFA/GGBS = 1:1; (**b**) MSWIFA/GGBS = 1:3.

Figure 6 presents XRD patterns of hardened pastes (7 days and 28 days of curing) incorporating different MSWIFA types and mix ratios. Key crystalline phases identified across all specimens include calcite (CaCO_3_), quartz (SiO_2_), NaCl, ettringite (AFt), and Friedel’s salt. The prominent CaCO_3_ peaks primarily originate from the MSWIFA feedstock, with potential minor contributions from carbonation. The characteristic broad amorphous hump centered around 29° (2θ, ~25–40°) corresponds to C-(A)-S-H gel [40].

RFA’s high chloride content manifests as intense NaCl and Friedel’s salt peaks (Figure 6a,b). Cl^−^ reacts with aluminates under alkaline activation to form Friedel’s salt. The prominent NaCl peak, primarily inherent to RFA with minor contribution from Cl^−^-NaOH reaction, diminishes by 28 days. This indicates Cl^−^ speciation shifts towards Friedel’s salt formation or adsorption onto C-(A)-S-H gel via van der Waals forces/electrostatic attraction over time.

The pyrolysis reduces the chloride content but increases the sulfate content in PFA/T-PFA, thereby altering the hydration reaction pathway. Sulfates, as the primary reactants for the formation of AFt [41], have a higher exothermicity than the formation of Friedel’s salts, which also explains the higher heat release observed in PFA/T-PFA (refer to Section 3.4). XRD patterns (Figure 6a,b) further show distinct peaks for AFt in both PFA and T-PFA, along with broad peaks for C-(A)-S-H. As the curing time increases, the intensity of the C-(A)-S-H broad peak strengthens, indicating the gradual formation of hydration products. Pyrolysis also enhances the solubility of sulfate compounds, while soluble chlorides are effectively removed, reducing the competition for Friedel’s salt formation. Meanwhile, during water soaking, metallic aluminum (Al^0^) and soluble aluminates are partially leached. During the alkali activation process, the released sulfate ions (SO_4_^2−^) and dissolved aluminate species (Al(OH)_4_^−^) react with calcium from GGBS in the high-pH environment, preferentially forming stable AFt [42,43]. Aluminum and sulfates are transformed into AFt, accompanied by the formation of C-A-S-H, which increases the density and strength of the microstructure. This process ultimately shifts the potential expansion mechanism to a performance-enhancing reaction. This has been supported by the literature on alkali-activated systems [44,45].

T-PFA exhibits more prominent AFt peaks than PFA, suggesting water-soaking promotes AFt formation. This is likely to be due to aluminum conversion during soaking (e.g., to soluble aluminates), enhancing its availability to react with sulfate and alkali to form AFt. No new crystalline phases appear, confirming water treatment modifies reactivity without altering fundamental reaction types. These hydration products (AFt, Friedel’s salt, C-(A)-S-H gel) play critical roles in HM stabilization/solidification (S/S).

**Figure 7:**
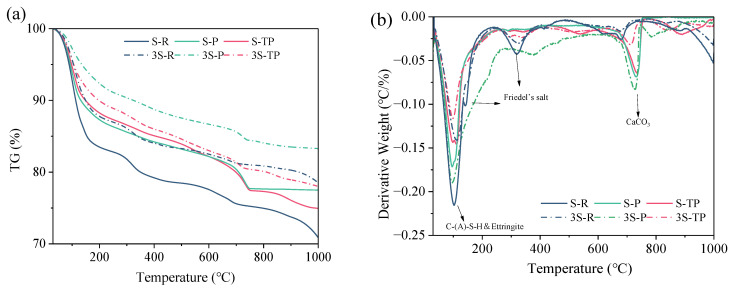
TGA curves of AAM paste specimens: (**a**) TG; (**b**) DTG.

Figure 7 presents TG and DTG curves for paste specimens. Specimens with a 1:3 MSWIFA/GGBS ratio consistently show lower total mass loss (30–1000 °C) than their 1:1 counterparts (Figure 7a), indicating lower bound water/hydration product content in higher-GGBS mixes. This mass loss arises from the decomposition of hydration products, the loss of free/adsorbed water from porous MSWIFA, and potential carbonation. DTG analysis (Figure 7b) identifies characteristic decomposition ranges: AFt dehydration (~100 °C), Friedel’s salt decomposition (~150 °C & 300 °C), CaCO_3_ decarbonation (~720 °C), and a broad 50–400 °C peak corresponding to the decomposition of C-(A)-S-H gel and other aluminosilicate hydrates [46].

Considering MSWIFA’s hygroscopicity and potential deliquescence, mass loss analysis focused on specific ranges [39,40]. Free and weakly bound water content can be evaluated at 50–200 °C, and the content of major hydration products (e.g., C-(A)-S-H gel, AFt) can be assessed at 200–400 °C.

**Figure 8:**
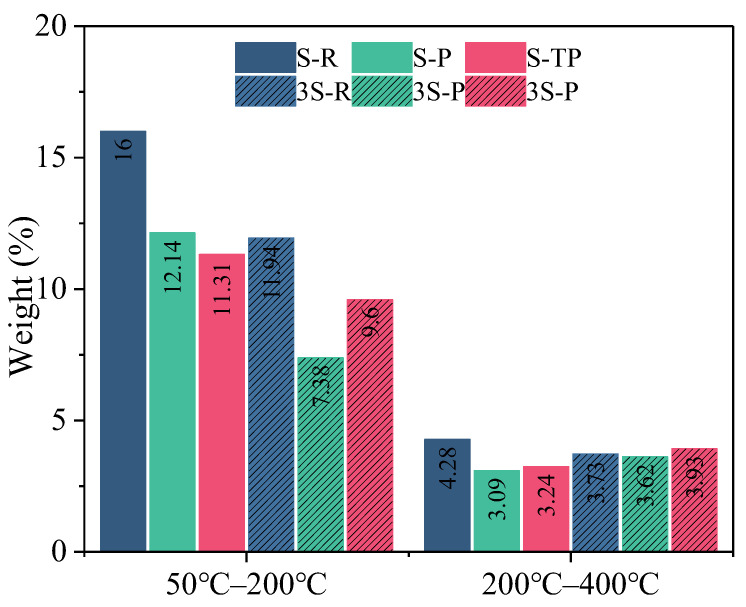
Mass loss rate in the 50–200 °C and 200–400 °C ranges for hardened pastes.

Figure 8 summarizes mass loss in these key ranges. When the temperature is between 50 and 200 °C, higher fly ash content (1:1 ratio) correlates with greater mass loss, indicating a lower hydration degree and higher residual free/weakly bound water. RFA exhibits the highest loss in this range. However, when the temperature is between 200 and 400 °C, lower fly ash content (1:3 ratio) generally corresponds to higher mass loss (except RFA), signifying the formation of more hydration products. Crucially, T-PFA shows higher mass loss than PFA within this range, confirming its enhanced hydration degree (consistent with strength and calorimetry results). RFA’s anomalously high mass loss here does not indicate more hydration products; rather, it reflects significant retention of weakly bound water due to its low reactivity and disrupted matrix.

### 3.6. Microstructural Analysis (SEM)

**Figure 9:**
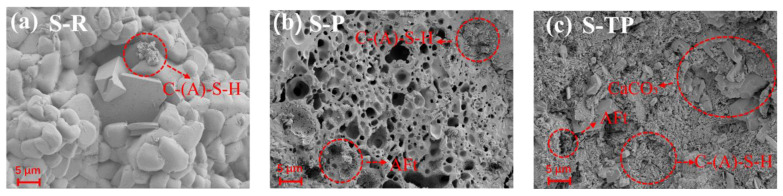
SEM micrographs of 28-day-cured AAM pastes (MSWIFA/GGBS = 1:1): (**a**) RFA-based (S-R); (**b**) PFA-based (S-P); (**c**) T-PFA-based (S-TP).

Figure 9 presents SEM micrographs of 28-day hardened pastes (MSWIFA/GGBS = 1:1) for all three fly ash types. As shown in Figure 9a, the matrix surface of RFA appears relatively clean and dense, with no obvious macropores. Minor quantities of C-(A)-S-H gel are visible. Friedel’s salt was not detected, consistent with its low formation level in RFA matrices. The PFA sample (Figure 9b) exhibits numerous irregularly sized pores distributed throughout the matrix. These pores result from hydrogen gas generation during the reaction between residual elemental aluminum (Al^0^) in PFA and the alkaline activator. Within the pores, the presence of ettringite (AFt) needles and C-(A)-S-H gel is evident. While these hydration products form within the void spaces, the overall pore structure increases matrix porosity. After water-soaking treatment, the T-PFA sample (Figure 9c) demonstrates a significantly refined microstructure compared to PFA. Large pores are markedly reduced, resulting in a visibly denser matrix. Abundant C-(A)-S-H gel, minor AFt, and calcite particles fill the microstructure. This enhanced compactness directly arises from water-soaking pretreatment effectively removing expansion-causing Al^0^.

The SEM observations corroborate compressive strength results: the elimination of Al^0^-induced pores and the formation of a dense, hydration-product-filled microstructure in T-PFA are key factors underlying its superior mechanical performance.

### 3.7. Pore Structure and Distribution (MIP)

**Figure 10:**
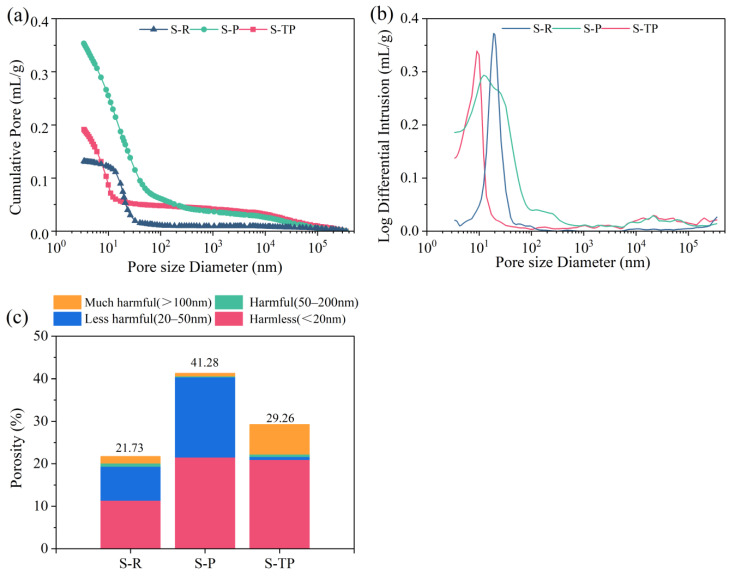
Pore structure of MSWIFA-AAM pastes: (**a**) cumulative intrusion; (**b**) log differential intrusion; (**c**) porosity and pore size distribution.

Figure 10 presents MIP results for 28-day-hardened pastes (MSWIFA/GGBS = 1:1). Pores were classified based on established criteria [47]: harmless (<20 nm), less harmful (20–50 nm), harmful (50–200 nm), and very harmful (>200 nm). Regarding the total porosity, it can be found that PFA had significantly increased total porosity (41.28%) compared to RFA (21.73%), while T-PFA had reduced porosity (29.26%) (Figure 10c), representing a 29.1% decrease relative to PFA.

Regarding the pore size distribution, it can be found that PFA exhibited a left-shifted, broader, and lower-amplitude PSD peak (Figure 10b), indicating a substantial increase in all pore size ranges. Notably, harmful and very harmful pores became dominant (Figure 10c). After water soaking, T-PFA showed a dramatic refinement in PSD. While the peak remained broadened, the critical shift was a massive increase in the proportion of harmless pores (<20 nm), reaching 71.5% (Figure 10c). Concurrently, the volume of harmful pores (>50 nm) significantly decreased. The high total porosity in PFA stems primarily from hydrogen gas generation due to residual elemental aluminum (Al^0^) reacting with the alkaline activator, creating large, interconnected voids (harmful/very harmful pores). Water soaking effectively removes Al^0^, eliminating the expansion mechanism. This allows the enhanced formation and denser packing of hydration products (confirmed by XRD/TG/DTG/SEM), converting large harmful pores into fine, harmless pores (<20 nm).

Although the RFA sample exhibited lower total porosity, its matrix comprised largely unreacted fly ash particles due to its low reactivity and insufficient hydration. This resulted in a weak framework potentially containing larger, interconnected cracks and voids that severely compromise structural integrity. In contrast, the T-PFA sample, despite its higher overall porosity, developed a robust, well-hydrated matrix. The porosity in T-PFA is predominantly composed of small, isolated gel pores within the abundant hydration products (C-A-S-H and AFt), which have a minimal negative impact on strength and effectively immobilize heavy metals. Despite higher total porosity than RFA, the refined pore structure (71.5% < 20 nm pores) in T-PFA is the primary factor enabling its superior compressive strength. Fine pores (<20 nm) contribute positively to strength via improved matrix packing density. While PFA’s increased reactivity promotes hydration product formation (explaining its strength gain over RFA), the detrimental effect of Al^0^-induced large pores (>50 nm) outweighs this benefit, limiting its strength potential and causing late-age reduction. T-PFA resolves this conflict by eliminating the pore-generating mechanism (Al^0^) while preserving high reactivity. As a result, water-soaking treatment of pyrolyzed fly ash achieves critical pore refinement—drastically increasing the proportion of strength-enhancing harmless pores (<20 nm) by eliminating Al^0^-induced expansion—resulting in a denser, more durable matrix with superior mechanical performance.

### 3.8. Heavy Metal Leaching Behavior

**Figure 11:**
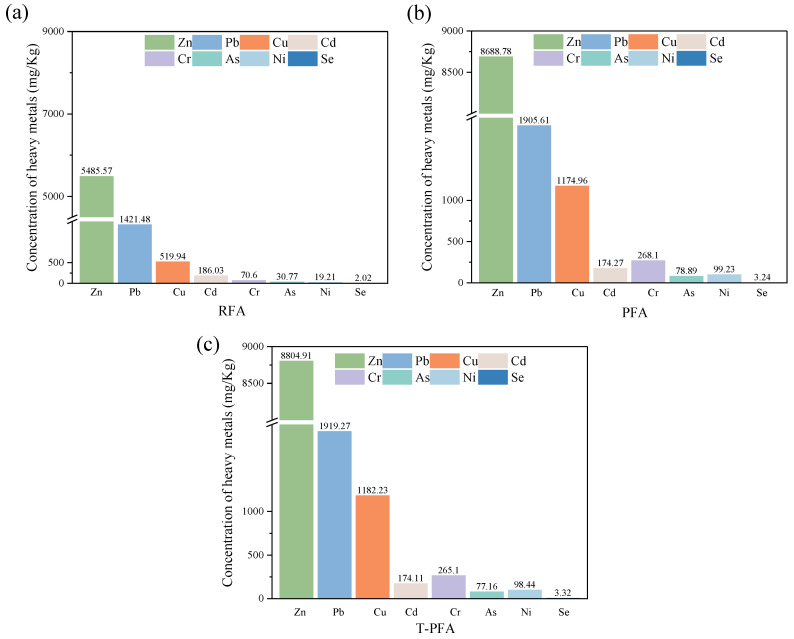
Total heavy metal concentrations in MSWIFA: (**a**) RFA; (**b**) PFA; (**c**) T-PFA.

Figure 11 presents acid digestion results quantifying the total HM content in RFA, PFA, and T-PFA. All samples contained detectable levels of Cd, Cu, Cr, Pb, and Zn (predominant HMs), with lower concentrations of Ni, As, and Se. Crucially, pyrolysis significantly increased total HM content in PFA and T-PFA compared to RFA (Figure 11a vs. Figure 11b,c); this is attributed to reductive reactions with carbon during pyrolysis concentrating metals in the ash matrix. T-PFA exhibited a near-identical HM content to PFA, confirming water soaking selectively removes elemental aluminum without extracting soluble salts or HMs.

**Figure 12:**
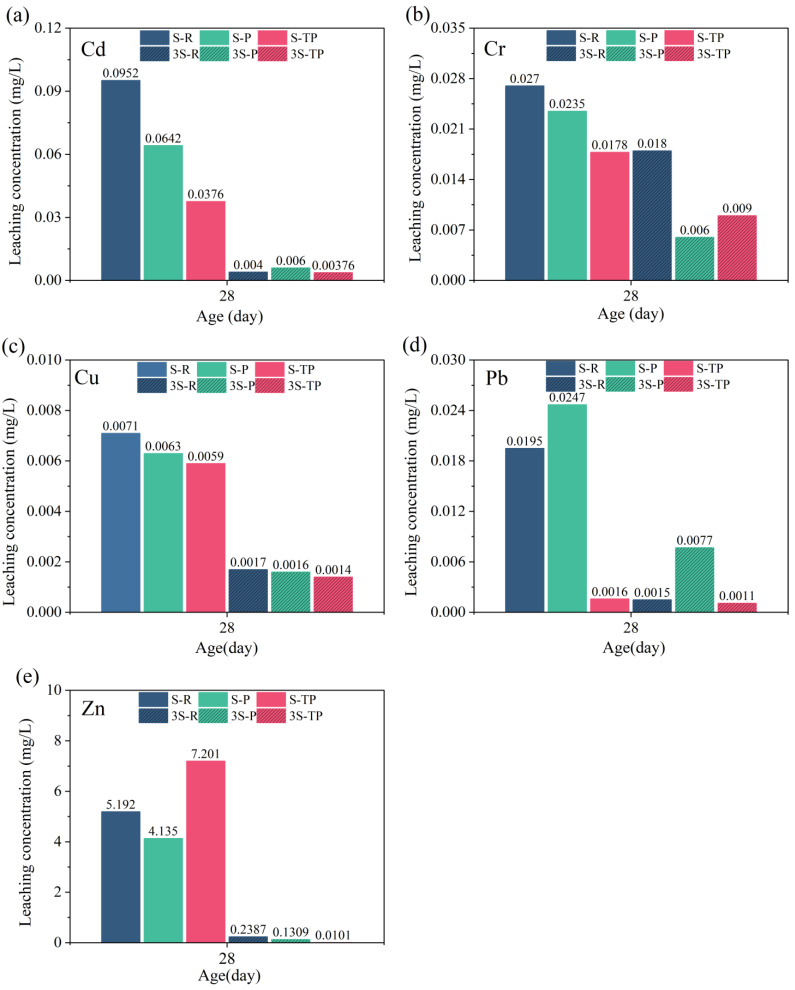
Leaching concentrations of Cr (**a**), Cd (**b**), Cu (**c**), Pb (**d**), and Zn (**e**) from hardened paste samples.

To evaluate solidification/stabilization (S/S) performance, three fly ash types at two MSWIFA/GGBS ratios were subjected to TCLP leaching tests after 28 days. The leaching concentrations of Cr, Cd, Cu, Pb, and Zn in hardened pastes (Figure 12) were compared with the limits specified in HJ 1134-2020 (Table 5). All values were below permissible thresholds. In general, specimens with a 1:3 ratio exhibited lower leaching concentrations than those with a 1:1 ratio, owing to the higher GGBS content and reduced MSWIFA proportion, which increased hydration product formation and produced a denser matrix. These products enhanced the S/S effect via adsorption, ion exchange, and physical encapsulation [3,48,49,50].

At 28 days, RFA specimens showed higher leaching of Cr, Cd, and Cu than PFA and T-PFA specimens, particularly at the 1:1 ratio. This is mainly due to RFA’s low reactivity and limited hydration, resulting in fewer hydration products to immobilize HMs. In addition, strength retrogression during curing increased internal porosity, weakened physical encapsulation, and accelerated HM migration. An excessive chloride content may further aggravate HM leaching [51].

Pyrolysis increased the reactive phase content in RFA, thereby promoting more hydration products and strengthening the matrix, which enhanced HM immobilization. However, Pb immobilization in PFA was poor at both ratios, with 28-day leaching concentrations exceeding those in RFA and T-PFA. This was likely caused by Al-induced expansion, which increased porosity and reduced encapsulation efficiency [52].

Water soaking removed expansive Al from PFA, producing a denser matrix and freeing activators to participate in hydration. This increased hydration degree and product content, further improving HM S/S. Consequently, most HMs in T-PFA specimens showed lower leaching concentrations than those in RFA and PFA specimens at both ratios. An exception occurred for Zn at the 1:1 ratio, where T-PFA exhibited higher leaching than RFA and PFA. This may be related to Zn’s amphoteric behavior: in highly alkaline environments, Zn can form soluble Zn(OH)_2_, increasing its mobility [53,54]. Nevertheless, Zn leaching remained more than 90% below the regulatory limit (HJ 1134-2020).

Overall, using PFA and T-PFA to prepare AAMs provides an effective S/S strategy for HMs in MSWIFA. Although pyrolysis increases the total HM content, the combined pyrolysis and water-soaking treatments enhance the reactive phase content, eliminate expansion, and maintain strength development, thereby densifying the matrix and improving immobilization via chemical adsorption, ion exchange, and physical encapsulation. While Pb mobility increased in high-porosity PFA and Zn mobility was slightly higher in alkaline T-PFA, all leachate concentrations were well within environmental standards, confirming the environmental safety and technical feasibility of this approach.

## 4. Conclusions

This study employs municipal solid waste incineration fly ash (MSWI FA) and ground granulated blast furnace slag (GGBS) to produce alkali-activated cementitious materials. The effects of different fly ash pretreatment methods—pyrolysis and pyrolysis followed by water soaking—and varying FA-to-GGBS ratios on mechanical properties, hydration kinetics, microstructural development, and heavy metal (HM) stabilization/solidification (S/S) were systematically investigated. The results show that pyrolysis combined with water soaking significantly enhances hydration reactivity and improves HM immobilization. Key findings are as follows:

(1) Pyrolysis increased the proportion of reactive phases in raw FA and reduced its Cl^−^ content, promoting hydration. Subsequent water soaking consumed the expansive metallic Al in the pyrolyzed FA without significantly altering its physicochemical composition, but it did result in a denser matrix and more complete utilization of activators in hydration. At an FA/GGBS ratio of 1:1, compressive strength at 56 days increased by 107.47% after pyrolysis and by 295.33% after pyrolysis plus water soaking, compared with the control.

(2) Pyrolysis removed Cl^−^ from raw FA and increased both the sulfate content and reactive phases, altering the hydration pathway. In addition to C-(A)-S-H gel, ettringite (AFt) was formed, while Friedel’s salt was absent, leading to a higher hydration degree than in untreated FA. Water soaking of pyrolyzed FA ensured that activators previously consumed by Al reactions were fully available for hydration, further increasing the reaction degree and hydration product yield compared with both raw and pyrolyzed FA.

(3) Binders containing pyrolyzed FA and water-soaked pyrolyzed FA consistently kept HM leachate concentrations below regulatory limits. Pyrolysis increased reactive phase content, promoting more hydration products (C-A-S-H gel and AFt) and maintaining higher matrix strength, thereby improving HM immobilization. Water soaking further densified the matrix, enhancing physical encapsulation of HMs. Overall, the combined pyrolysis–water-soaking treatment of FA in alkali-activated binders ensures both technical performance and environmental safety.

## 5. Future Directions

This study uses pyrolysis and water-soaking treatments on fly ash to enhance its performance, primarily focusing on mechanical properties and heavy metal stability. Although the method has been proven feasible, it also has some limitations. For example, due to time constraints, the study could not perform repeated tests on fly ash from different regions or batches. Additionally, there is a lack of durability-related testing, and the migration risk of heavy metals in the solidified body under long-term or extreme environmental conditions has not been assessed. Future research could focus on these areas. Moreover, although the study analyzed the microstructure, it did not conduct an in-depth quantitative analysis of the hydration products and structures observed in the SEM images. Furthermore, computer vision and deep learning techniques offer considerable potential for automatically quantifying microstructural features—such as pores, cracks, and hydration products—in SEM images, thereby enabling more objective and efficient microstructural analysis and durability performance evaluation. Robust and efficient vision-based models, such as DeepLab [55] and EfficientNet [56], could provide innovative solutions and new research directions for this type of analysis.

## Figures and Tables

**Table 1:** Chemical compositions of GGBS, PFA, and RFA (wt.%).

Oxides	SiO_2_	CaO	Al_2_O_3_	MgO	SO_3_	TiO_2_	Fe_2_O_3_	Na_2_O	K_2_O	Cl	ZnO	Others
GGBS	31.04	40.21	15.94	7.70	2.28	0.88	0.32	-	-	-	-	0.58
PFA	12.48	46.59	5.94	3.72	17.57	1.40	4.40	0.81	0.93	1.34	1.59	3.3
RFA	3.50	40.48	1.04	0.84	7.69	0.18	0.73	13.13	7.24	23.68	0.65	0.84

**Table 2:** The TOC test results for RFA and PFA (wt.%).

Types of Fly Ash	TC	IC	TOC
RFA	4.883	3.254	1.629
PFA	2.529	1.909	0.62

**Table 3:** The table of comparative mix design of AAM specimens (kg/m^3^).

Mix ID	RFA	PFA	T-PFA	GGBS	Na_2_SiO_3_	NaOH	Water
S-R	175.8	-	-	175.8	57.8	16.5	92.2
S-P	-	175.8	-	175.8			
S-TP	-	-	178.5	175.8			
3S-R	89.7-	-		263.7			
3S-P	-	89.7	-	263.7			
3S-TP	-	-	89.7	263.7			

**Table 4:** Chemical compositions of PFA and T-PFA (wt.%).

Oxides	SiO_2_	CaO	Al_2_O_3_	MgO	SO_3_	TiO_2_	Fe_2_O_3_	Na_2_O	K_2_O	Cl	ZnO	Others
PFA	12.48	46.59	5.94	3.72	17.57	1.40	4.40	0.81	0.93	1.34	1.59	3.3
T-PFA	11.85	46.61	5.70	3.46	19.21	1.31	4.38	0.84	0.89	1.14	1.46	3.15

**Table 5:** The limits of HM TCLP test results of MSWIFA samples (mg/L).

HMs	Cu	Cd	Cr	Pb	Zn
Limits	40	0.15	0.5	0.25	100

## Data Availability

The original contributions presented in this study are included in the article. Further inquiries can be directed to the corresponding author.
